# Sodium 4-Phenylbutyrate Protects Hypoxic-Ischemic Brain Injury via Attenuating Endoplasmic Reticulum Stress in Neonatal Rats

**DOI:** 10.3389/fnbeh.2021.632143

**Published:** 2021-02-11

**Authors:** Ziyi Wu, Jiayuan Niu, Hang Xue, Shuo Wang, Ping Zhao

**Affiliations:** Department of Anesthesiology, Shengjing Hospital of China Medical University, Shenyang, China

**Keywords:** sodium 4-phenylbutyrate, endoplasmic reticulum stress, hypoxic-ischemic brain injury, CREB, learning and memory

## Abstract

Neonatal hypoxic-ischemic (HI) brain injury is associated with long-term neurological disorders, and protective strategies are presently scarce. Sodium 4-phenylbutyrate (4-PBA) reportedly acts as a chemical chaperone that alleviates endoplasmic reticulum (ER) stress, which plays a critical role in neurological diseases. The present study aimed to evaluate the neuroprotective effects of 4-PBA on HI-induced neonatal brain injury in a rat model, and to characterize possible underlying mechanisms. The HI brain injury model was established by ligating the left common carotid artery in 7-day-old rats, followed by exposure to 8% oxygen for 2 h. The 4-PBA or vehicle was administered by an intracerebroventricular injection 30 min before HI. The protein expression levels of ER stress markers (GRP78, ATF6, and CHOP) were detected by western blotting at 24 h after HI insult. The activation of cAMP-response element-binding protein (CREB) was evaluated by western blotting and immunofluorescence. TUNEL and Nissl staining were performed to detect the histomorphological changes in the hippocampal neurons at 24 h and 7 days, respectively, after HI injury. From days 29 to 34 after brain HI, rats underwent Morris water maze tests to assess cognitive functioning. The results showed that pretreatment with 4-PBA decreased HI-induced excessive ER stress and neuronal injury. Moreover, CREB activation might be involved in the beneficial effects of 4-PBA on HI-induced learning and memory deficits in rats. In conclusion, the present study suggested a potential therapeutic approach of ER stress inhibition in the treatment of neonatal HI brain injury.

## Introduction

Perinatal hypoxic-ischemic (HI) is one of the leading causes of brain injury in neonates, and which may be associated with subsequent neurological disabilities such as cerebral palsy, mental retardation, and epilepsy (Douglas-Escobar and Weiss, 2015). Although some promising neuroprotective agents have been used for potential neuroprotection against neonatal HI-induced brain injury (Wu et al., 2015), clinically practical methods have not been well-established. Therefore, the identification of novel therapeutic targets based on mechanistic studies is still urgently needed.

The endoplasmic reticulum (ER) is the cellular organelle that is responsible for protein folding and secretion, calcium homeostasis, and lipid biosynthesis. A wide variety of pathophysiological and pharmacological insults can disturb proper ER function and thereby lead to an accumulation of misfolded and unfolded proteins in the ER (Schwarz and Blower, 2016). Prolonged expression of misfolded/unfolded proteins triggers ER stress, which initiates a cascade of reactions called the unfolded protein response (UPR) (Oakes and Papa, 2015). The UPR is an adaptive survival mechanism that orchestrates the recovery of ER homeostasis by attenuating protein translation, enhancing degradation of misfolded proteins, and inducing ER-resident chaperones, e.g., glucose-regulated protein 78 (GRP78) (Oakes and Papa, 2015). However, under sustained or severe ER stress conditions, cells may ultimately undergo irreversible damage and apoptotic cell death. ER stress has therefore been implicated in the pathophysiology of several neurodegenerative diseases, such as Alzheimer’s disease, Parkinson’s disease, and stroke (Ghemrawi and Khair, 2020). Thus, therapeutic interventions targeting ER stress are supposed to be a potential therapy for neonatal HI brain injury.

Sodium 4-phenylbutyrate (4-PBA) is a low molecular weight fatty acid, which has been approved for clinical use to treat urea cycle disorders (Kolb et al., 2015). In addition, 4-PBA acts as a chemical chaperone to alleviate ER stress by assisting proper posttranslational modification and folding, to reduce the load of mutant or mislocated proteins retained in the ER lumen (Yam et al., 2007; Mizukami et al., 2010; Yang et al., 2016). However, the neuroprotective effect of 4-PBA with respect to inhibition of ER stress in neonatal HI brain injury has not been characterized.

Emerging evidence suggests that excessive ER stress may exert adverse effects on brain development and contribute to long-term cognitive impairments (Xu et al., 2015). The cAMP-response element binding protein (CREB), a ubiquitously expressed transcription factor, has been implicated in the regulation of learning and memory function (Saura and Cardinaux, 2017). Inactivation of CREB leads to transcription suppression of synaptic proteins and neurotrophic factors. We therefore aimed to investigate whether 4-PBA pretreatment protected against brain damage by attenuating ER stress following HI in neonatal rats. Furthermore, we examined whether CREB activation played an essential role in the beneficial effects of 4-PBA on neurobehavioral performance.

## Materials and Methods

### Animals and Study Groups

Seven-day-old Sprague-Dawley rat pups were obtained from the Benxi Base for Medical and Pharmaceutical Research and Education, Shengjing Hospital (Liaoning, China). All animal experiments were in accordance with the principles outlined in the National Institute of Health Guideline for the Care and Use of Laboratory Animals and approved by the Institutional Animal Care and Use Committee of Shengjing Hospital, China Medical University (No. 2017PS020K). Every effort was made to minimize animal suffering and reduce the number of animals used in the experiments. A total number of 120 rat pups were randomly divided into three groups (*n* = 40, male/female ratio, 1:1) involving the sham, HI, and HI + 4-PBA groups.

### The Neonatal HI Injury Model and Intracerebroventricular Injection

The neonatal HI injury model was established as previously described (Zhao et al., 2007). Briefly, rat pups were anesthetized with isoflurane and subjected to permanent double ligation of the left common carotid artery using 7-0 surgical silk. The surgery was completed in ≤5 min. After recovering from anesthesia, the pups were sent back to their dams for 2 h. The pups were then placed in a chamber with an atmosphere of 8% O_2_ balanced with 92% N_2_ for 2 h at a continuous air temperature of 37°C.

The pups received an intracerebroventricular injection, which was performed at the left lateral ventricle (coordinates: 2 mm posterior, 1.5 mm lateral, 3 mm below the skull surface) as described previously (Ma et al., 2016). The 4-PBA (200 μM, 2 μL, SML0309; Sigma-Aldrich, St. Louis, MO, United States) or the corresponding volume of vehicle was injected 30 min before HI.

### Mortality, Body Weight Monitoring, and Brain Loss Quantification

Death was recorded during the period from the onset of cerebral HI to 34 days afterward, followed by calculation of the mortality rate. Body weights were measured just before and on day 7 and on day 34 after HI. Pups (*n* = 10, per group) were sacrificed at 7 days after the brain HI, and the two cerebral hemispheres of the brain were separated and weighed. The weight ratios of left to right hemispheres were then calculated.

### Western Blotting

At 24 h after treatment, the left cerebral hippocampus of five pups were collected. Protein extraction was conducted by homogenization in RIPA lysis buffer (Beyotime Institute of Biotechnology, Jiangsu, China) containing protease and phosphatase inhibitors (Roche, Germany), with further centrifugation at 14,000 rpm for 30 min at 4°C. The supernatants were collected and the protein concentration was measured using the BCA Protein Assay Kit (Beyotime Institute of Biotechnology, China). Equivalent amounts of protein were separated by SDS-PAGE gels and then transferred onto polyvinylidene difluoride membranes (Millipore, Darmstadt, Germany). After blocking with 5% bovine serum albumin for 2 h at room temperature, the membranes were incubated with the primary antibodies overnight at 4°C. The primary antibodies were anti-GRP78 (1:500; Abcam, Cambridge, United Kingdom), anti-CHOP (1:1,000; Cell Signaling Technology, Danvers, MA, United States), anti-CREB (1:1,000; Cell Signaling Technology), and anti-p-CREB (1:1,000, Cell Signaling Technology). Next, the membranes were incubated with secondary antibodies (Zsbio, Beijing, China) for 2 h at room temperature. The protein blots were detected and photographed using a GE Amersham Imager 600 (Little Chalfont, United Kingdom) using a SuperSignal^®^ West Pico Chemiluminescent Substrate (Thermo Fisher Scientific, Waltham, MA, United States).

### Immunofluorescence Staining

Deeply anesthetized pups (*n* = 5, per group) were transcardially perfused with saline followed by 4% phosphate-buffered paraformaldehyde (PFA) 24 h after treatment. Neonatal brains were immediately removed and postfixed in 4% PFA overnight at 4°C, then embedded in paraffin after dehydration in an ethanol gradient. Three micron thick coronal sections were made, and then were deparaffinized and heated in citrate buffer for antigen retrieval. Phosphate-buffered saline (PBS) containing 10% fetal bovine serum was added to each section for 30 min at room temperature to reduce background staining. Next, the sections were incubated with p-CREB antibody (1:100; Cell Signaling Technology) overnight at 4°C in a humidified chamber. The next day, the sections were incubated with secondary antibody for 2 h at room temperature. The nuclei were stained with 4′,6-diamidino-2-phenylindole (DAPI). Sections were then photographed using a Nikon Ni-U microscope (Nikon, Tokyo, Japan) by an investigator who was blinded to the experimental interventions. Three fields of vision in the hippocampal CA1 region of the left cerebral hemispheres were randomly selected per section to take photographs and for cell counting. Quantification was performed using NIS-Elements AR Analysis 4.50.00 software and then we calculated the ratio of the number of p-CREB positive cells to the total number of cells.

### Terminal Deoxynucleotidyl Transferase dUTP Nick End-Labeling (TUNEL) Staining

The TUNEL assay was performed on 3-μm thick paraffin-embedded sections using the Cell Death Detection Kit (Roche, Penzberg, Germany). The brain sections (*n* = 5, per group) were deparaffinized and heated in citrate buffer for antigen retrieval. PBS containing 10% fetal bovine serum was added to each section for 30 min at room temperature to reduce background staining. Next, the sections were incubated with terminal deoxynucleotidyl transferase and dUTP for 1 h at 37°C. Nuclei were stained with DAPI. Three fields of vision in the hippocampal CA1 region of the left cerebral hemispheres were randomly selected per section and photographed using a Nikon C1 microscope by an investigator who was blinded to the experimental interventions. The number of TUNEL-positive cells was counted by NIS-Elements AR Analysis 4.50.00 software. The index of apoptosis was calculated as the ratio of the overall number of apoptotic cells to the total number of cells.

### Nissl Staining

We performed histopathological evaluation of pup brains, 7 days after HI-induced brain injury (*n* = 5, per group). Coronal sections (4 μm thick) approximately 3.5 mm caudal to bregma were obtained for Nissl staining. An observer who was blinded to the group assignments examined these sections. The neuronal density in the CA1 region of the left cerebral hemispheres was determined by counting the number of cells that were positive for Nissl staining in a reticle (approximately 0.034 mm^2^) with Image J software (National Institutes of Health, Bethesda, MD, United States). Images of cells from three sections were calculated in each rat.

### Morris Water Maze Tests

Morris water maze tests were conducted from days 29 to 34 after brain HI to test the learning and memory abilities. The water maze consisted of a round pool (160 cm wide and 60 cm high) with black walls. The pool was filled with water to a depth of 30 cm at 20°C, with a 12 cm wide cylindrical platform set 1.5 cm below the water surface and 30 cm away from the wall in the target quadrant. During the navigation test, training sessions lasted for 5 days and began at 8:00 am every day, four times daily, with an interval of 30 min. Rats (*n* = 10, per group) were placed in the water to search for the underwater platform from four quadrants facing the pool wall. If successful in locating the platform within 90 s, the rats were made to stay on the platform for 20 s. If the rats did not find the platform within 90 s, they were guided to the platform to stay on it for 20 s, and the escape latency was recorded as 90 s. A spatial probe test was conducted at 24 h after the last training session. Rats were placed in the water and allowed to freely swim for 90 s after the platform was removed. The swimming routes and navigation positioning strategy were analyzed using a video tracking system (Shanghai Mobile Datum Ltd., China).

### Statistical Analysis

All the data were analyzed using SPSS statistical software for Windows, version 19.0 (SPSS, Chicago, IL, United States). The data are shown as the mean ± SD. Comparisons between groups were analyzed by one-way analysis of variance and the Student-Newman-Keuls method. The escape latency was analyzed using two-way analysis of variance with repeated measures followed by the Bonferroni *post hoc* test. The spatial probe test data were analyzed using the Kruskal–Wallis with Dunn’s multiple comparison test. The mortality was analyzed by *Z* testing. A *p*-value < 0.05 was considered significant.

## Results

The general characteristics of each group are shown in Table 1. The mortality of the groups after HI-induced brain injury was approximately 8%, and was not significantly decreased with 4-PBA pretreatment. There was no difference in body weights, either before or on day 7, or on day 34 after HI, among the different groups.

**TABLE 1:** General characteristics.

	**Fate after brain Hypoxic-ischemia (HI)**			**Body weight (g) of the survivors**		
	**Total**	**Dead**	**Mortality %**	**7 days old**	**7 days after HI**	**34 days after HI**
Sham	50	0	0	13.9 ± 1.6	35.8 ± 0.9	143.7 ± 13.2
HI	50	4	8	14.2 ± 1.3	33.7 ± 1.4	139.6 ± 11.7
HI + 4PBA	50	3	6	14.1 ± 1.8	34.1 ± 1.7	140.9 ± 10.1

*Values (body weight) are mean ± SD; Sham, sham surgery; HI, hypoxic-ischemia.*

### The 4-PBA Inhibited ER Stress-Related Protein Expression at 24 h After HI in Neonatal Rats

**FIGURE 1:**
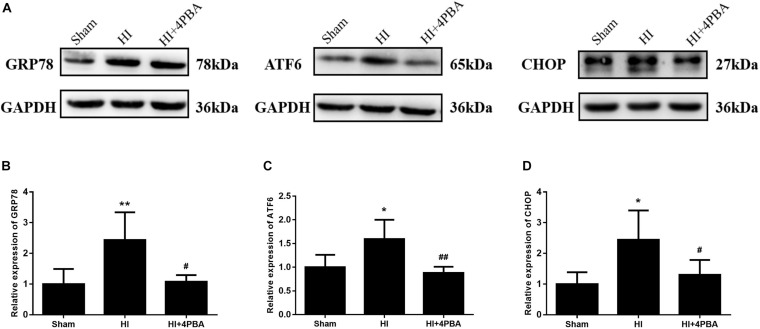
Sodium 4-phenylbutyrate (4-PBA) alleviated ER stress at 24 h after neonatal hypoxic-ischemic (HI) in the neonatal rat brain. **(A)** Representative western blotting images. **(B)** Quantitative evaluation of GRP78. Sham: (1 ± 0.4901), HI: (2.438 ± 0.9015), HI + 4-PBA: (1.072 ± 0.2196). **(C)** Quantitative evaluation of ATF6. Sham: (1 ± 0.5356), HI: (1.788 ± 0.4528), HI + 4-PBA: (0.984 ± 0.1475). **(D)** Quantitative evaluation of CHOP. Sham: (1 ± 0.3849), HI: (2.446 ± 0.9524), HI + 4-PBA: (1.297 ± 0.4879). Glyceraldehyde 3-phosphate dehydrogenase was used as an internal standard. Values are presented as the mean ± SD (*n* = 5); *p < 0.05; **p < 0.01, compared with the sham group; ^#^*p* < 0.05; ^##^*p* < 0.01, compared with the HI group.

To investigate the effects of 4-PBA on ER stress, ER stress proteins were measured by western blotting. GRP78, ATF6, and CHOP are commonly used as markers of ER stress (Zheng et al., 2014). Compared with the sham group, protein expression levels of GRP78, ATF6, and CHOP in the ipsilateral hippocampus were significantly upregulated at 24 h following HI (Figure 1; *p* < 0.01, *p* < 0.05, *p* < 0.05, respectively), and were significantly decreased by 4-PBA administration (Figure 1; all, *p* < 0.05). These data indicated that ER stress was excessively activated in the hippocampus of rats after HI, and 4-PBA could effectively alleviate the ER stress.

### The 4-PBA Attenuated Neuronal Apoptosis at 24 h After HI in Neonatal Rats

**FIGURE 2:**
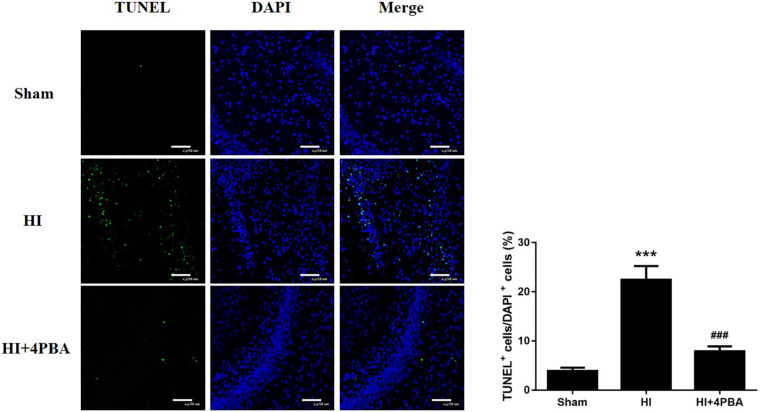
Sodium 4-phenylbutyrate (4-PBA) attenuated neuronal apoptosis at 24 h after neonatal hypoxic-ischemic (HI) in the neonatal rat brain. **Left**, representative images of TUNEL-stained cells in the CA1 region of the left hippocampus. The TUNEL signal is shown in fluorescent green and the stained nuclei are shown in fluorescent blue [4′,6-diamidino-2-phenylindole (DAPI)]. Scale bar = 50 μm. **Right**, the quantitative analysis results of the TUNEL stain signals. The percentage of TUNEL-positive cells was expressed as the number of TUNEL-stained nuclei divided by the total number of DAPI-stained nuclei. Sham: (3.88 ± 0.687), HI: (22.46 ± 2.784), HI + 4-PBA: (7.88 ± 0.9985). Values are presented as the mean ± SD (*n* = 5); ***p < 0.001, compared with the sham group; ^###^*p* < 0.001, compared with the HI group.

Persistent and unresolved ER stress can trigger apoptotic cell death (Faitova et al., 2006). To determine whether neuroprotection from 4-PBA was associated with anti-apoptotic activity, neuronal apoptosis was evaluated using the TUNEL assay. As shown in Figure 2, increased TUNEL-positive neurons were detected in the left hippocampal CA1 region 24 h after HI (Figure 2; *p* < 0.001). In addition, pretreatment with 4-PBA significantly decreased TUNEL-positive neurons compared with the HI group in the corresponding brain sections (Figure 2; *p* < 0.001).

### The 4-PBA Reduced Brain Tissue Loss and Neuronal Loss at 7 Days After HI

**FIGURE 3:**
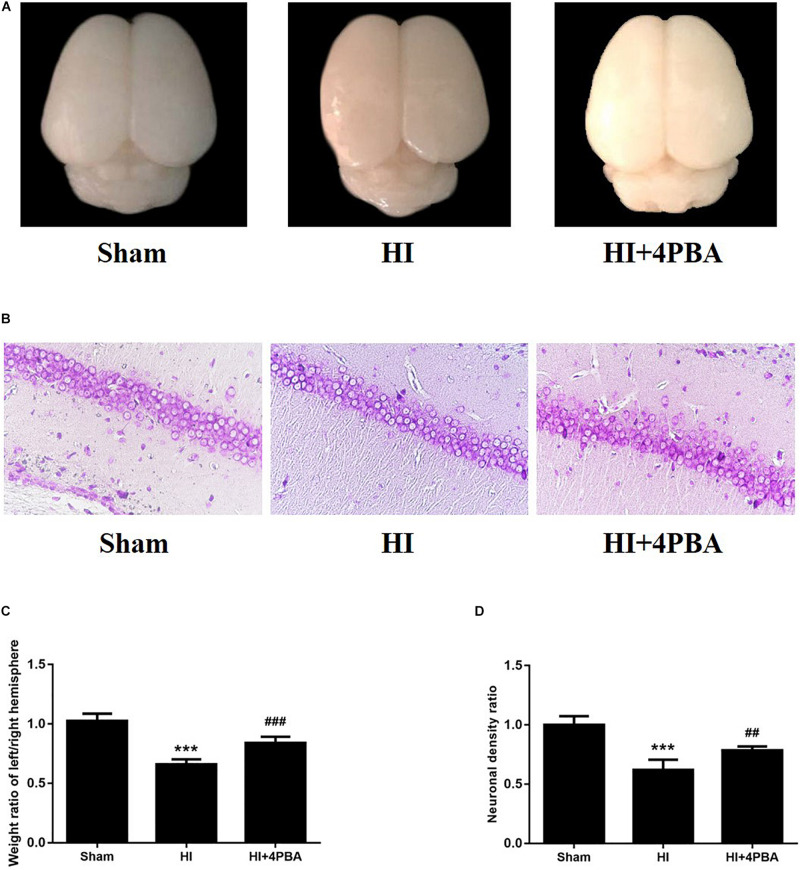
Brain weight ratio and brain histopathology analysis of the pups 7 days after neonatal hypoxic-ischemic (HI). **(A)** Representative images of cerebral morphology. **(B)** Quantitative analysis of weight ratio of left/right hemisphere. Sham: (1.025 ± 0.0603), HI: (0.6581 ± 0.044), HI + 4-PBA: (0.8394 ± 0.0530). Values are the mean ± SD (*n* = 10); ***p < 0.001, compared with the sham group, ^###^*p* < 0.001, compared with HI group. **(C)** Representative images of the histopathological Nissl staining in the CA1 of the left hippocampus. **(D)** Quantitative analysis of neuronal density ratios (left/right) in the CA1 region of the hippocampus in pups. Sham: (1 ± 0.0726), HI: (0.618 ± 0.0882), HI + 4-PBA: (0.7846 ± 0.0335). Values are presented as the mean ± SD (*n* = 10); ***p < 0.001, compared with sham group; ^##^*p* < 0.01, compared with HI group.

To examine whether 4-PBA reduced HI-induced brain tissue loss, the right and left cerebral hemispheres of rats were harvested and weighed at 7 days after the brain HI. As shown in Figure 3, HI led to a significant tissue loss of the left cerebral hemisphere compared with the sham group (Figures 3A,C; *p* < 0.001), while such loss was attenuated by 4-PBA pretreatment (Figures 3A,C; *p* < 0.001).

In addition, HI injury in neonatal rats induced losses of neurons in hippocampal CA1 regions of the left cerebral hemispheres, as shown by fewer Nissl-staining positive cells and decreased neuronal density ratios (Figures 3B,D; *p* < 0.001). Treatment with 4-PBA prior to HI induction increased the number of Nissl-staining positive cells and neuronal density ratios (Figures 3B,D; *p* < 0.01).

### The 4-PBA Rescued HI-Induced Spatial Learning and Memory Deficits

**FIGURE 4:**
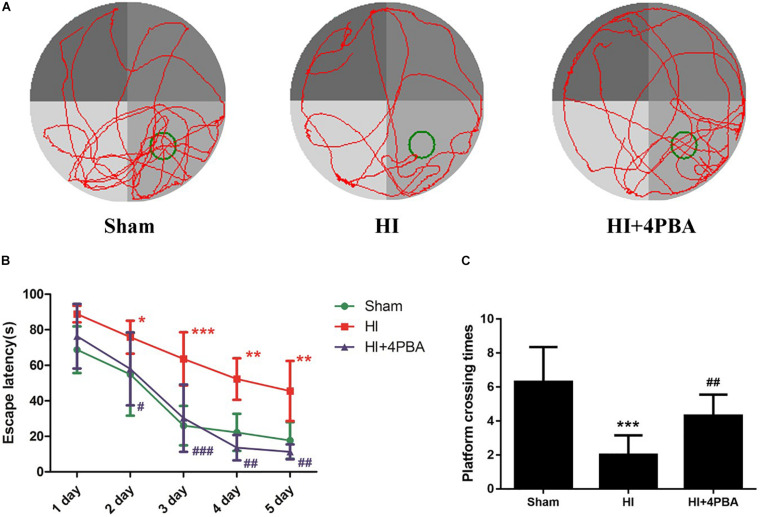
4-PBA improved learning and memory ability in the Morris water maze tests. **(A)** The display of tracks of all groups during the probe trials. **(B)** Escape latency. **(C)** Platform crossing time. Sham: (6.3 ± 2.058), neonatal hypoxic-ischemic (HI): (2 ± 1.155), HI + 4-PBA: (4.3 ± 1.252). Values are presented as the mean ± SD (*n* = 10); *p < 0.05; **p < 0.01; ***p < 0.001 compared with sham group; ^#^*p* < 0.05; ^##^*p* < 0.01; ^###^*p* < 0.001 compared with the HI group.

Morris water maze tests were used to detect learning and memory functions. There was no significant difference in swimming speed (data not shown). As shown in Figure 4, during the navigation test, compared with the sham group, the rats in the HI group had longer escape latencies to reach the hidden platform (Figure 4B; day 2, *p* < 0.05; day 3, *p* < 0.001; day 4, *p* < 0.01; and day 5, *p* < 0.01). However, the use of 4-PBA significantly shortened the latency periods after HI (Figure 4B; day 2, *p* < 0.05; day 3, *p* < 0.001; day 4, *p* < 0.01; and day 5, *p* < 0.01). Using the spatial probe test, the platform crossing times in the HI group were decreased compared with the sham group (Figure 4C; *p* < 0.001), and the decrease was attenuated by pretreatment with 4-PBA (Figure 4C; *p* < 0.01).

### The 4-PBA Increased p-CREB Protein Expression After HI

**FIGURE 5:**
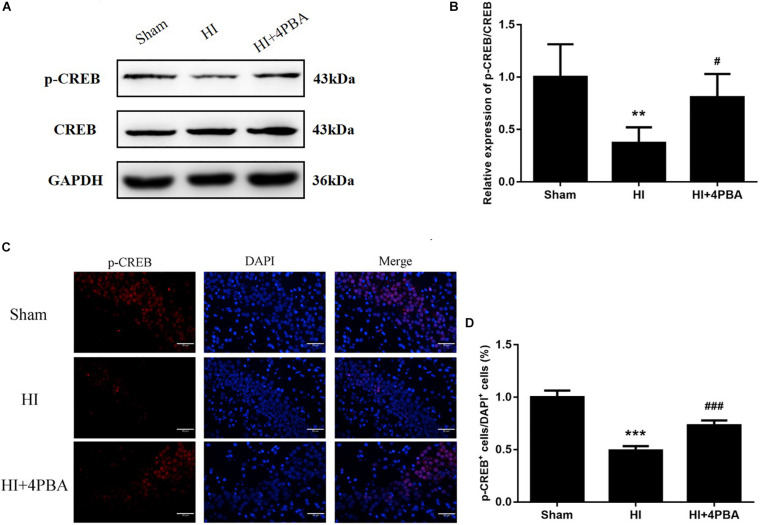
Sodium 4-phenylbutyrate (4-PBA)increased p-CREB protein expression at 24 h after neonatal hypoxic-ischemic (HI) in the neonatal rat brain. **(A)** Representative western blotting images. **(B)** Quantitative evaluation of the levels of p-CREB/CREB. Values are the mean ± SD (*n* = 5). Sham: (1 ± 0.3127), HI: (0.372 ± 0.1484), HI + 4-PBA: (0.8079 ± 0.221). **(C)** Fluorescent staining of p-CREB in the CA1 region of the left hippocampus. Scale bar = 50 μm. **(D)** The quantitative analysis result of the p-CREB stain. Values are presented as the mean ± SD (*n* = 5). Sham: (1 ± 0.0628), HI: (0.4916 ± 0.0417), HI + 4-PBA: (0.7303 ± 0.0473); **p < 0.01; ***p < 0.001, compared with the sham group; ^#^*p* < 0.05; ^###^*p* < 0.001, compared with the HI group.

cAMP-response element-binding protein, a critical element modulating long-term learning and memory, was tested by western blotting and immunofluorescence staining to identify possible mechanisms for the long-term neuroprotection by 4-PBA administration in neonatal rats with HI. Compared with the sham group, as shown in Figure 5, the protein expression of p-CREB was downregulated after HI (Figures 5A,B; *p* < 0.05). Moreover, the downregulation was significantly attenuated by 4-PBA treatment (Figures 5A,B; *p* < 0.05).

These findings were consistent with immunofluorescence results, because decreased p-CREB positive cells were observed in the hippocampal CA1 region of the left cerebral hemispheres after HI, while 4-PBA administration partly reversed this result in the same region (Figure 5C). Taken together, the results suggested that 4-PBA rescue of learning and memory deficits may have involved activation of CREB.

## Discussion

Neonatal HI brain injury is a leading cause of lifelong neurological impairment. As a result, there is an urgent need for the development of effective drugs or strategies for this devastating disease. In the present study, we showed the potent neuroprotective potential of 4-PBA in a neonatal rat model of HI. Seven-day-old neonatal rats subjected to unilateral common carotid arterial ligation followed by exposure to 8% oxygen for different durations provide the most widely used animal model of neonatal HI brain injury (Yager and Ashwal, 2009). Our study showed that HI increased numerous ER stress markers, including GRP78, activating transcription factor 6 (ATF6), and CCAAT/enhancer binding protein homologous protein (CHOP), in the hippocampus of neonatal rats. Strikingly, treatment with 4-PBA, a known ER stress inhibitor, not only suppressed ER stress, but also reduced neuronal apoptosis and brain tissue loss. Furthermore, 4-PBA treatment alleviated long-term spatial learning and memory impairments in rats, possibly, mediated by CREB activation.

Accumulating evidence has shown that ER stress is implicated in the physiopathology of several neurological disorders, such as Alzheimer’s disease, Parkinson’s disease, and amyotrophic lateral sclerosis (Cabral-Miranda and Hetz, 2018). ER stress can be triggered by multiple stimuli and pathological conditions, such as hypoxia, ischemia, oxidative injury, and inflammation, which leads to accumulation of misfolded and unfolded proteins (Chi et al., 2019). ER stress activates a group of signal transduction pathways called the UPR to restore ER homeostasis, promoting cell survival and adaptation. However, under unresolvable ER stress conditions, the UPR promotes apoptosis. To investigate ER stress following HI treatment of rats, we examined the expression levels of specific hallmarks of ER stress, including GRP78, ATF6, and CHOP. GRP78 is a resident protein of the ER and upon ER stress, it first dissociates from ER membranes to initiate the UPR and to decrease protein refolding and degradation (Ibrahim et al., 2019). ATF6, one of the so-called ER stress sensors, plays a central role in the initiation and regulation of the UPR (Tam et al., 2018). Prolonged and/or serious ER stress elicits apoptotic signals via CHOP, a key mediator of ER stress-induced apoptosis (Li et al., 2014). In this study, upregulation of the protein expression of GRP78, ATF6, and CHOP, paralleled with increased apoptotic cells in the hippocampus of rats, supported the possibility that neonatal cerebral HI injury might induce apoptotic neuronal death in the hippocampus through induction of the ER stress pathway.

As small molecular factors, chemical chaperones have emerged as a novel therapeutic approach against neurological disorders, by promoting ER folding (Engin and Hotamisligil, 2010). 4-PBA is a low molecular weight non-specific chemical chaperone known to stabilize protein conformations, assist protein normal folding, and improve the traffic of aberrant proteins, thus relieving ER stress. It has been shown to ameliorate cerebral ischemia (Qi et al., 2004; Yang et al., 2017), spinal cord ischemia (Mizukami et al., 2010), and Parkinson’s disease (Inden et al., 2007) in animal models. In the present study, treatment with 4-PBA significantly attenuated HI-induced ER stress in neonatal rats, as shown by a significant reduction in levels of ER stress-related proteins. 4-PBA administration was associated to decreased TUNEL-positive cells and increased Nissl-positive cells in the hippocampus, suggesting it had beneficial effects for attenuating ER stress-induced apoptosis and neuronal loss. Moreover, HI impaired long-term spatial-dependent learning and memory dysfunction in rats, which was attenuated by 4-PBA.

A further study was conducted to identify possible mechanisms for the long-term neuroprotection by 4-PBA administration after HI in neonatal rats. It is well-established that CREB may be a universal modulator of processes required for a variety of complex forms of memory, including spatial-dependent learning and memory (Restivo et al., 2009). Previous studies showed that the expression of p-CREB in rats with memory impairment is significantly decreased (Xiang et al., 2019). In the present study, the expression of p-CREB was significantly decreased in the HI group, when compared with the sham group. Inactivation of CREB plays an essential role for ER stress-induced synaptic and memory dysfunction. In the current study, 4-PBA administration attenuated HI-induced downregulation of p-CREB levels in the hippocampus.

## Conclusion

Hypoxic-ischemic in neonatal rats induced excessive ER stress, likely involving ER stress-mediated cell death, which aggravated brain injury and induced cognitive and memory impairments during adolescence by reducing the levels of p-CREB. 4-PBA administration partially reversed the results by inhibiting ER stress. The use of 4-PBA in the immature brain to treat HI brain injury creates new opportunities for clinical protective strategies in the future.

## Data Availability Statement

The raw data supporting the conclusions of this article will be made available by the authors, without undue reservation.

## Ethics Statement

The animal study was reviewed and approved by the Institutional Animal Care and Use Committee of Shengjing Hospital, China Medical University (Approval No. 2017PS020K).

## Author Contributions

JN and PZ conceived and designed the experiments. HX, JN, and SW performed the experiments, and generated and analyzed the data. ZW wrote the manuscript. All authors read and approved the final manuscript.

## Conflict of Interest

The authors declare that the research was conducted in the absence of any commercial or financial relationships that could be construed as a potential conflict of interest.
